# The Association between Participation in Fights and Bullying and the Perception of School, Teachers, and Peers among School-Age Children in Serbia

**DOI:** 10.3390/children9010116

**Published:** 2022-01-17

**Authors:** Sanja Stankovic, Milena Santric-Milicevic, Dejan Nikolic, Nenad Bjelica, Uros Babic, Ljiljana Rakic, Zorica Terzic-Supic, Jovana Todorovic

**Affiliations:** 1 Centre for Biochemistry Clinical Laboratories, University Clinical Centre of Serbia, 11000 Belgrade, Serbia; sanjast2013@gmail.com; 2 Institute for Social Medicine, Faculty of Medicine, University of Belgrade, 11000 Belgrade, Serbia; zoricaterzic37@gmail.com (Z.T.-S.); jovana.todorovic@med.bg.ac.rs (J.T.); 3 Center-School of Public Health and Health Management, Faculty of Medicine, University of Belgrade, Pasterova 2, 11000 Belgrade, Serbia; 4 Faculty of Medicine, University of Belgrade, 11000 Belgrade, Serbia; denikol27@gmail.com (D.N.); uros.babic@med.bg.ac.rs (U.B.); 5 Physical Medicine and Rehabilitation Department, University Children’s Hospital, 11000 Belgrade, Serbia; 6 Primary Health Care Centre “Dr Simo Milosevic”, 11000 Belgrade, Serbia; n.bjelica74@gmail.com; 7 University Clinical Centre of Serbia, Faculty of Medicine, University of Belgrade, 11000 Belgrade, Serbia; 8 Clinic for Hematology, University Clinical Centre of Serbia, 11000 Belgrade, Serbia; lj.rakic68@gmail.com

**Keywords:** fighting, bullying, school-age children, school, relations, professors, Serbia

## Abstract

Participating in physical fighting and bullying can be a cause of severe injury and death among school-age children. Research evidence can support school and health actors’ efforts to improve school-age children’s development and health capacity for life. The study aims to assess the prevalence of school-age children’s participation in fights and bullying in Serbia, and to examine the relevance of students’ socio-demographic characteristics and perceptions of school and relations with other students and professors for participation in fights and bullying. A secondary analysis is also performed on the original data of the 2017 HBSC study, which was conducted on 3267 students in a nationally representative sample of primary and high schools in Serbia. We sought to investigate the relationship between eight socio-demographic characteristics and nine school-related perceptions, with two outcome variables: taking part in fights and taking part in bullying, examined by using univariate and multivariate logistic regression. The main results show that 50.8% of boys and 17.1% of girls have taken part in fights, while 17.7% boys and 10.4% of girls have taken part in bullying. Students who felt a large and very large burden of school obligations were 1.43 times more likely to participate in bullying at least once, while they were 1.38 and 2.12 times more likely to participate in multiple fights and 4.04, 1.24, and 2.78 times more likely to participate multiple times in bullying. Multiple participation in fights and in bullying is significantly negatively associated with female gender, younger age years, good and very good perception of family financial status and quality of life, and positive perceptions of school and relations they have with other students and professors. Fights among school-age children are significantly positively associated with living with relatives/legal guardians and poor quality of life. In conclusion, the prevalence of participating in at least one fight/bullying is higher than in multiple fights/bullying. These associations suggest a necessity to enhance the monitoring and control of peer behavior among school-age children. The findings of the study imply key enablers of protection, such as building relationships based on team spirit and work, friendly behavior, empathy, and help, which should be included in the value system of school and family activities in programs to combat fights and bullying in school-age children.

## 1. Introduction

The health and development of school-age children is a contemporary topic of various health policies and programs at global [1,2], regional [3], and national [4] levels, which has become even more of a focus in critical situations such as the COVID-19 pandemic [5]. The recent global estimates on adolescents in Europe pointed out that injuries are the major causes of morbidity and premature mortality, causing 1466.7 and 2979.0 DALYs per 100,000 in age groups 10–14 years and 15–19 years, respectively [6]. School authorities pay special attention to the development of school-age children by preventing them from participating in peer-fights and bullying. Moreover, UNICEF monitors bullying in order to mobilize support systems to increase adolescent protection around the world [7]. Crucial for reducing bullying and fights among school-age children are the United Nations Sustainable Development Goals, targets 16.2, 5.2, 10.3, and 11.7, which explicitly refer to reducing the physical and psychological violence among youth, gender inequality, discriminatory policies, and the lack of safe spaces [8]. Due to the negative reflections of bullying on academic achievement, self-esteem, motivation, and cognitive aspects [9,10], the UNESCO Institute for Statistics (UIS) publishes data on the strengths and weaknesses of school systems [11]. The latter source indicates that the percentage of students in primary and lower secondary education experiencing bullying in the last 12 months varies across countries, and in 2019, it ranged from 33.17% for females and 45.85% for males (Armenia) to 96.43% for females and 97.50% for males (Philippines) [11]. The study that was performed in low- and middle-income countries of different WHO regions found the male gender was stressed to be more frequently involved versus females regarding injuries, physical attacks and physical fighting [12]. By the UIS datastores [11], that prevalence in Serbia was also higher among boys than girls and has declined for the five-year period from 62.06% (in 2015) to 56.82% (in 2019) for boys and from 53.69% (in 2015) to 46.82% (in 2019) for girls. Given that almost one in two children of school-age were involved in bullying, it would be important to analyze their perceptions of school and their relations with peers and teachers. While fighters do not see avoiding fights as an effective conflict resolution strategy [13], it is very important to understand the protective influence of school settings as well. From qualitative research, it is known that anger and a lack of effective conflict-resolution skills, combined with poor emotional regulation, can easily led to aggression [14]. Belonging to gangs might provide strength to those students who need it, and school can be a place to show off their superiority by engaging in physical fights and bullying “opponents”. The risk of bullying and fights could be lowered by preventive measures and actions that favor positive peer influence and peer support during adolescence [15,16] and in school settings in particular [14,17]. Differences in the prevalence of bullying and its types along with better understanding of such phenomena in school-age children can have an impact on various authorities, including schools, parents or legal guardians, and health care workers, to propose and create preventive measures and interventions that would have a positive response in reducing unfavorable behavioral patterns. Therefore, understanding school-related contexts for participation in fights and bullying among school-age children is important to prevent this violent behavior from escalating and to make schools a safe and secure environment for children’s development and education.

The study aims to assess the prevalence of the participation of school-age children (11–17 years old) in fights and bullying in Serbia. In addition, the study examines the relevance of students’ socio-demographic characteristics and perceptions of school, other students, and professors for participation in fights and bullying using a nationally representative sample of primary and high schools. The study’s findings could inform policymakers and decision-makers who want to improve school safety and youth health and promote factors that have the potential to protect school-age children from participating in fights and violence.

## 2. Methods

### 2.1. Study Design and Sample

In order to explain participating in fights and bullying among school-age children in Serbia, we conducted a secondary analysis of the original data of the 2017 HBSC study taken from the Institute of Public Health of Serbia (IPHS)’s electronic database. In the 2017 HBSC study in Serbia, the nationally representative sample consisted of 3267 students (51.3% were boys and 48.7% girls) from 64 primary (grades V and VII) and high schools (grade I of gymnasium and secondary vocational school) on the territory of the Republic of Serbia (not including data for Kosovo and Metohija) [18].

The sample’s representativeness at the national level was guaranteed for primary and secondary schools in all four administrative regions (Vojvodina, Belgrade, Southern and Eastern Serbia, and Sumadija and Western Serbia). The IPHS created a sampling algorithm to determine the probability proportional to a school’s size (by 1500 children of a given grade) and a random number of classes in each school. When the main sample did not reach the expected number of participants, the school replacements were activated.

The IPHS, in cooperation with the World Health Organization (WHO); the Serbian Ministry of Education, Science, and Technological Development; and the Ministry of Health, conducted the 2017 HBSC study and stored the original data [18]. The IPHS used the WHO’s methodology and the standardized international research protocol and HBSC standardized questionnaire for the 2017 HBSC study [18]. According to the HBSC protocol [18], the schools’ stakeholders were informed on the research aim, protocol, and ethics. The HBSC data in the IPHS database were collected upon schools giving their consent to participate and obtaining parental approval for children’s voluntary and anonymous participation in the Serbian HBSC study.

This secondary data analysis of the 2017 HBSC study was approved by the Ethics Committee of IPHS (Decision 1934/1, 3 March 2020).

### 2.2. Study Variables

The study explored three sets of variables of school-age children, including (1) a set of eight socio-demographic characteristics, (2) a set of nine school-related perceptions, and (3) taking part in fights and in bullying. This study has two outcome variables: taking part in fights and taking part in bullying. The socio-demographic variables and school-related perceptions of students were examined as explanatory variables of partaking in fights and partaking in bullying.

The socio-demographic characteristic are gender (boys; girls), age (11–12 years old; 13–14 years old; 15–17 years old); school grade (grade V of primary school; grade VII of primary school; grade I of high school); family size (2–3; 4–5; 6–7; 8 and more members in the family), living with (both parents, one parent, one parent and stepfather/mother, relatives/legal guardians), having brothers/sisters (none; one sister/brother; and two or more sisters/brothers), perception of family financial status (bad; average; good), and quality of life perception (very bad; bad; average; good; very good).

The studied student perceptions related to school, other students, and professors include the following:

- What do you think about school? (I don’t like school at all/too much; I love school to some extent; I love school very much).
- Are school obligations a big burden for you? (not at all/yes, a little; yes, enough; yes, very much).
- How do you think your class teacher evaluates your success in relation to others in the class? (below average/average; good; very good).
- Students in my class like to be together (disagree; neither agree nor disagree; agree).
- Most of the students in my class are friendly and want to help (disagree; neither agree nor disagree; agree).
- Other students accept me as I am (disagree; neither agree nor disagree; agree).
- I feel that the professors accept me as I am (disagree; neither agree nor disagree; agree).
- I feel that professors take care of me as a person (disagree; neither agree nor disagree; agree).
- I have great confidence in my professors (disagree; neither agree nor disagree; agree).

Taking part in fights and bullying was investigated with the following questions [18]: “How many times have you participated in a fight during the last 12 months?” and “How often have you been involved in abusing another student during the last few months?”. Students’ response categories were: none (I have not participated), at least once (once/once or twice), and multiple times (more than once/twice and more) and “I don’t know/no answer”—missing. The response rate by variables ranged from 92.6% to 98.8% so that the overall musings in responses were less than 8%.

### 2.3. Statistical Analysis

We explored the prevalence (percentages with a 95% confidence interval—CI) of outcome variables and assessed the statistically significant different categorical variables (at *p* < 0.05) with the Pearson chi-square test.

The study has examined four models of participating in fights and bullying, including:

- Model 1: Participating in fight at least once versus none;
- Model 2: Participating in fight multiple times versus none;
- Model 3: Participating in bullying at least once versus none;
- Model 4: Participating in bullying multiple times versus none.

The models quantified the strength of the correlation between the explanatory variables and four outcome variables by using a cross-odds ratio (OR) with a 95% confidence interval (CI) of the univariate and multivariate logistic regression. All statistical analyses were performed by the SPSS software package, version 22.0 (2018).

## 3. Results

### 3.1. Prevalence of Participating in Fights in Serbia

In Serbia, the prevalence of participating in fights was almost three times higher among school-age boys than girls (Table 1), i.e., almost every second boy and every sixth girl participated in fights (50.8% versus 17.1%, respectively, *p* < 0.001). The prevalence of participating in at least one fight is higher than in multiple fights and is significantly higher among boys than girls (at least one fight: 33.4% versus 13.4%, respectively; and multiple fights: 17.4% versus 3.6%, respectively).

Partaking in fighting was most prevalent among school-age children between 13–14 years old and in grade VII of primary school, with more than one third of all of them taking part in fights. While the highest participation in at least one fight was among those aged 13–14 years old (28.4%, *p* < 0.001) and in grade VII of primary school (28.0%, *p* < 0.001), the prevalence of multiple fighting was the highest among students at aged 15–17 years old (12.7%, *p* = 0.005) and at grade I of high school (12.7%, *p* = 0.004).

The prevalence of participating in fights also varies in regard to some family characteristics; almost every second student with very bad quality of life perception (50.0%, *p* = 0.019) and bad perception of family financial status (43.2%, *p* = 0.046) was fighting. While there is no significant difference in terms of participating in a fight at least once, multiple-time involvement in fights was most prevalent in group of participants with bad perception of family financial status (19.4%, *p* = 0.002) and with very bad quality of life perception (33.3%, *p* < 0.001) (Table 1).

### 3.2. Prevalence of Participating in Bullying in Serbia

The prevalence of participation in bullying of school-age children (Table 2) was not as high as participation in fighting, but bullying was also more prevalent among school-age boys than girls (17.7% versus 10.4%, respectively, *p* < 0.001). The prevalence of participating in at least one act of bullying was higher than in multiple acts of bullying and was significantly more present among boys than girls (at least one bullying: 9.8% versus 7.3%, respectively, *p* = 0.01; and multiple bullying: 7.9% versus 3.1%, respectively, *p* < 0.001).

Partaking in bullying was most prevalent among school-age children of 13–14 years old (18.5%, *p* < 0.001) and in grade VII of primary school (18.2%, *p* < 0.001), i.e., approximately one in five of these students participated in the bullying. While the highest participation in at least one act of bullying was among those aged 13–14 years old (12.4%, *p* < 0.001) and in grade VII of primary school (12.2%, *p* < 0.001), the prevalence of partaking in multiple acts of bullying was the highest among students aged 15–17 years old and was almost equal among those of 13–14 years of age (6.3% and 6.2%, respectively, *p* = 0.026).

A total of 26.9% of students with a bad perception of family financial status (*p* < 0.001) and 41.4% of students with a very bad perception of quality of life participated in bullying (*p* < 0.001). Participation in bullying at least once was most common among students who live with one parent and stepfather/mother (16.9%, *p* = 0.026) and average quality of life perception (11.5%, *p* = 0.002). Involvement in bullying multiple times was most prevalent in the group of participants with bad perception of family financial status (16.0%, *p* < 0.001) and with very bad quality of life perception (34.5%, *p* < 0.001) (Table 2).

### 3.3. Model 1: Participating in Fights at Least Once versus None

According to univariate regression analysis, some of the students’ variables were significantly associated with participating in fighting at least once (Model 1) including gender, age, school type, liking the school, feeling cared for by professors, and having great confidence in professors (Table 3).

Multivariate regression analysis indicated that girls and high school students were less likely to take part at least once in fighting by 77% and 78%, respectively, while school-age children between 13–14 years were 3.15 times more likely to participate in fighting at least once. Some likely protective effects from participating in fights at least once included loving school to some extent (less likelihood by 25%), loving school very much (less likelihood by 57%), and having a great confidence in professors (less likelihood by 30%) (Table 3).

### 3.4. Model 2: Participating in Fights Multiple Times versus None

According to univariate regression analysis, multiple participation in fights (Model 2) was significantly associated with gender, age years, school type, living with relatives/legal guardians, perception of family financial status, quality of life, and perceptions of school, other students, and professors (Table 3).

Multivariate regression analysis indicated that students who felt a large and very large burden of school obligations were 1.38 and 2.12 times more likely to participate in multiple fights. Girls and students perceiving their quality of life as good and very good were less likely to take part in multiple fights by 88%, 76%, and 79%, respectively. Students who loved school to some extent and loved school very much were less likely by 49% and 68%, respectively, to take part in multiple fights. Multiple fighting was less likely among students who neither agreed nor disagreed as well as those who agreed that most of the students in class are friendly and want to help (by 42% and 56%, respectively). Similarly, participating multiple times in fighting was less likely among students who neither agreed nor disagreed as well as those who agreed that professors accept them as they are (by 48% and 59%, respectively). Those who neither agreed nor disagreed and those who agreed that they have great confidence in their professors were less likely by 39% and 56%, respectively, to participate in fighting multiple times (Table 3).

### 3.5. Model 3: Participating in Bullying at Least Once versus None

According to univariate regression analysis, the students’ variables significantly associated with participating in bullying at least once (Model 3) included gender, age years, school type, living with one parent and stepfather/mother, and perceptions of school, other students, and professors (Table 4).

Multivariate regression analysis indicated that girls as well as those who agreed that most students in class were friendly and wanted to help and those who agreed that professors take care of them were 36%, 27%, and 47% less likely to take part in at least one act of bullying, respectively. Similarly, those who neither agreed nor disagreed and those who agreed that they have great confidence in their professors were less likely by 32% and 41%, respectively, to participate in multiple acts of bullying. However, school-age children of 13–14 years old and those living with one parent and stepfather/mother were 3.26 times and 2.35 times, respectively, more likely to participate in bullying at least once. Furthermore, students who felt a large and very large burden of school obligations were 1.41 and 1.43 times more likely to participate in bullying at least once.

### 3.6. Model 4: Participating in Bullying Multiple Times versus None

According to univariate regression analysis, multiple participation in bullying (Model 4) is significantly associated with gender, age years, school type, perception of family financial status, quality of life, and perceptions of school, other students, and professors (Table 4).

Multivariate regression analysis indicated that students aged 13–14 years old and those who felt a large and very large burden of school obligations were 4.04, 1.24, and 2.78 times more likely to participate multiple times in bullying. Girls and students perceiving average and good family financial status and average, good, and very good quality of life were less likely to take part multiple times in bullying by 62%, 42%, 61%, and 85%, 87%, and 89%, respectively. Students who thought that their class teacher evaluates their success as good and very good in relation to others in the class were less likely by 45% and 51%, respectively, to take part in multiple bullying. Multiple acts of bullying were less likely among students who neither agreed nor disagreed and those who agreed that most of the students in class are friendly and want to help (by 47% and 71%, respectively). Similarly, they were likely among students who neither agreed nor disagreed and those who agreed that professors accept them as they are (by 49% and 58%, respectively). Those who neither agreed nor disagreed and those who agreed that they have great confidence in their professors were less likely to participate multiple times in bullying by 52% and 55%, respectively (Table 4).

## 4. Discussion

This study on a Serbian representative sample showed that participation in fights and participation in bullying was more frequent in boys versus girls. School peers were more prone to involvement in fights than participation in bullying. We have demonstrated that every second boy participated in fights compared to less than every fifth girl, while less than every fifth boy and every tenth girl participated in bullying. Additionally, for both participation in fights and participation in bullying, a higher prevalence was seen for involvement at least once versus multiple involvements. Reports from the East Mediterranean region indicated that the prevalence of fights among boys was from 46–65%, while for girls it was lower, ranging from 21–48% [19,20]. Compared to this, a lower prevalence for boys was observed in the USA (39%), while for girls it was 23% [20]. Moreover, in a sample of Slovak adolescents, it was shown that boys were more prevalent in fight participation, with a higher prevalence of one-time participation [21]. Our study also demonstrated a higher prevalence of boys in participation of fights and less prevalent participation in multiple fights. Regarding participation in bullying, in the study of Carlyle and Steinman, it was noted that boys were more frequently the perpetrators [22]. Juvonen and Graham in their Review pointed out that in Western nations, 4–9% of youths frequently present with bullying behavior [23]. Aside from the more prevalent male gender in bullying participation, our study pointed out that the prevalence was above 10% for both boys and girls.

Bullying in youth can be considered a multidimensional complex social aspect. Previous reports have stated that bullies do not necessarily lack social skills nor the ability to regulate their emotions. It has also been stated that bullies have tendencies for domination and achievement of higher social status [23]. However, there is still open debate regarding factors influencing bullying behavior, including environmental and developmental changes in children. Having said this, our results point out that children aged 13–14 years old more frequently participated in both observed variables, where more than every third child participated in fights and less than every fifth child participated in bullying. The lower prevalence for both fifth graders of primary school and first graders of high school versus those from grade VII of primary school could point to the possible role of both aspects (environmental and developmental, for example: pubertal), since transitioning to a new school and new peers reduced the prevalence of both participation in fights and participation in bullying.

Considering the perception of family financial status, our study results demonstrated that less than every second child with bad perception and every third child with good perception participated in fights, while every fourth child with bad perception and almost every seventh with good perception participated in bullying. These findings clearly stress that as the perception of family financial status increases towards a more positive one, there is a decrease in participation in both fights and bullying of children in such families. Previously reported studies are not yet conclusive regarding the relationship between socioeconomic status and being a perpetrator for bullying in youth. In the meta-analysis of Tippett and Wolke [24], it was shown that bullying perpetration in the group of children and adolescents appeared through all socioeconomic categories, while in the study of Magklara et al., [25], the authors stated that lower socioeconomic status was associated with an increased risk of bullying perpetration in youth.

Compared to the family financial status, a similar trend can be observed for the perception of quality of life in our study, where every second child with very bad perception of quality of life and almost every third child with very good perception participated in fights, while less then every second child with very bad perception and every tenth child with very good perception participated in bullying.

In previous reports, it was noticed that several characteristics regarding teachers might be associated with peer victimization. Among them are the perception of problematic students’ behavior referred to internal and external causes, teachers’ beliefs that they can handle the bullying among students, and teachers’ history of bullying [26]. Furthermore, positive relations between teachers and students were shown to be associated with less bullying and fighting [27] as well as positive school climate with reduced bullying behavior in schools [28]. Our results are in line to a certain degree with previous reports, where a positive perception of school (students who like being at school) was a negative predictor for fighting participation. Even though our findings stress negative association between bullying and such positive perception of school, multivariate analysis revealed that this parameter is not a significant predictor. Contrary to the positive perception of school in terms of being in favor of attending one, we have noticed that students having a positive relation with their teacher in terms of fairness of evaluation is a negative predictor for bullying. Moreover, students’ positive perception of acceptance by and confidence in professors were shown to be negative predictors of fights and bullying in our study.

Our study confirmed that having friends is an essential protective factor from fighting and bullying in class [29]. Our study findings demonstrated that having friendships and classmates willing to help each other in the class, being fond of spending time together in class, and believing that one is accepted as an individual by classmates are associated with less likelihood for participation in fights multiple times and participation in bullying both once and multiple times. Friendships help students face problems by building their sense of belonging, self-esteem, and social skills [30] and defend them from bullying [31], highlighting the finding in our study that friendship among students in class and willingness to help each other is the predictor for less participation in fights multiple times and participation in bullying at least once. Participating in bullying and fighting affects everyone who is involved and witnesses it. Therefore, nurturing classmates’ empathy and friendships through skill-building for communication and relationships, promoting listening, self-reflection, self-improvement, and respect, is necessary to address aggressive social behavior among school-age children.

The present differences of the obtained results for fighting and bullying could suggest that potential intrinsic factors could play certain role in the determination of problematic behavior patterns. Additionally, these findings point out how complex and multidimensional the relations are between students and teachers as well as between peers that could have potential in the determination of unfavorable behavior. However, further studies are needed.

## 5. Study Limitations

Given the study’s limitations, this study allows for international comparisons and good practice learning if caution is taken in interpreting the findings. The main study limitation is that the survey question was about participation in bullying or fights. Therefore, it is impossible to distinguish the roles of school-age children in these conflicts based on this question alone. While psychological and other literature can shed light on the understanding and building of the mental capacities of the victims, perpetrators, witnesses, advocates, beneficiaries, etc., in this study, we analyzed children who participate in bullying and fights to distinguish the factors of emotionally charged relationships with school, teachers, and peers.

The study uses regression modeling among many variables from the HBSC study conducted at one point in time in Serbia to identify the leading associations between the school-age children’s attitudes toward school, teachers, and peers and violent behavior such as bullying and fighting. Therefore, both the modeling and the cross-sectional design explain the strong positive or negative relationship between input and outcome variables rather than determining the causal factors of bullying and fighting. Apart from the aspects observed in this study, other school-related aspects (such as school policies, resources, or programs) need to be explored. Therefore, there is room for qualitative research that could further help develop a deeper understanding of the profile of perpetrators of violence and the potential of protection mechanisms among school-age children. Although the study uses a nationally representative sample of schools, the study’s findings should not be generalized to school-age children outside Serbia due to community specifics.

Nonetheless, the sample type and size in the study show good correspondence with HBSC studies done in many other settings [32], allowing for creating joint anti-bullying and fighting programs of activities for school-age children at the regional level. At the local level, the study findings indicate the need to build teamwork and friendly relationships among students and facilitate the creation of procedures and programs that encourage supportive behavior and facilitate the respect of children and teachers’ right to learn in a safe and peaceful environment. The study’s findings indicate that schools in Serbia should cultivate schooling without violence and develop gender-designed specific skill-building interventions targeting school-age children prone to partaking in violent behavior against their peers.

## 6. Conclusions

In Serbia, every second boy participated in fights compared to less than every fifth girl, while less than every fifth boy and every tenth girl participated in bullying. Fights among school-age children are significantly positively associated with living with relatives/legal guardians and poor quality of life. In conclusion, the prevalence of participating in at least one fight/bullying is higher than in multiple fights/bullying. These associations suggest a necessity to enhance monitoring and control of peer behavior among school-age children. The findings of the study imply key enablers of protection such as building relationships based on team spirit and work, friendly behavior, empathy, and help, which should be included in the value system of school and family activities in programs to combat fights and bullying in school-age children.

## Figures and Tables

**Table 1:** Prevalence of participating in fights among school-age children in Serbia, 2017.

Variables	Sample * n (%)	Participation in Fights Prevalence (% and 95% Confidence Interval)		
		At Least Once	Multiple Times	Total
**Socio-Demographic Characteristics**				
**Gender**				
Boys	1654 (51.3)	33.4 (31.1–35.6)	17.4 (15.6–19.2)	50.8 (48.4–53.2)
Girls	1569 (48.7)	13.4 (11.8–15.1)	3.6 (2.7–4.6)	17.1 (15.2–18.9)
Pearson Chi-square *p*	0.134	<0.001	<0.001	<0.001
**Age (years)**				
11–12	873 (27.1)	23.1 (20.3–25.9)	8.8 (6.9–10.7)	32.0 (28.9–35.1)
13–14	939 (29.1)	28.4 (25.5–31.3)	9.5 (7.6–11.4)	37.9 (34.8–41.0)
15–17	1411 (43.8)	20.8 (25.5–31.3)	12.7 (10.9–14.4)	33.5 (31.1–36.0)
Pearson Chi-square *p*	<0.001	<0.001	0.005	0.019
**School type: grade**				
Primary: grade V	898 (27.9)	23.8 (21.0–26.6)	8.9 (7.0–10.8)	32.7 (29.7–35.8)
Primary: grade VII	928 (28.8)	28.0 (25.1–30.9)	9.4 (7.5–11.3)	37.4 (34.3–40.5)
High school: grade I	1397 (43.3)	21.0 (18.9–23.2)	12.7 (11.0–14.5)	33.8 (31.3–36.3)
Pearson Chi-square *p*	<0.001	<0.001	0.004	0.083
**Family size**				
2–3 members	392 (13.0)	20.9 (16.9–24.9)	11.5 (8.3–14.6)	32.4 (27.8–37.0)
4–5 members	1774 (58.6)	24.1 (22.1–26.1)	9.3 (7.9–10.7)	33.4 (31.2–35.6)
6–7 members	763 (25.2)	23.7 (20.7–26.7)	10.1 (8.0–12.2)	33.8 (30.5–37.2)
8 and more members	97 (3.2)	22.7 (14.3–31.0)	15.5 (8.3–22.7)	38.1 (28.5–47.8)
Pearson Chi-square *p*	<0.001	0.609	0.161	0.753
**Living with**				
Both parents	2558 (84.5)	23.2 (21.6–24.9)	9.8 (8.6–10.9)	33.0 (31.2–34.8)
One parent	374 (124)	24.9 (20.5–29.2)	9.9 (6.9–12.9)	34.8 (29.9–39.6)
One parent and stepfather/mother	59 (1.9)	28.8 (17.3–40.4)	11.9 (3.6–20.1)	40.7 (28.1–53.2)
Relatives/legal guardians	35 (1.2)	22.9 (8.9–36.8)	22.9 (8.9–36.8)	45.7 (29.2–62.2)
Pearson Chi-square *p*	<0.001	0.698	0.078	0.234
**Having brothers and sisters**				
None	352 (11.5)	23.9 (19.4–28.3)	10.5 (7.3–13.7)	34.4 (29.4–39.3)
One	1706 (55.5)	23.4 (21.4–25.4)	10.0 (8.5–11.4)	33.4 (31.1–35.6)
Two or more	1015 (33.0)	23.9 (21.3–26.6)	10.0 (8.2–11.9)	34.0 (31.1–36.9)
Pearson Chi-square *p*	<0.001	0.942	0.953	0.904
**Family financial status (perception)**				
Bad	155 (4.9)	23.9 (17.2–30.6)	19.4 (13.1–25.6)	43.2 (35.4–51.0)
Average	995 (31.2)	24.4 (21.8–27.1)	9.9 (8.1–11.8)	34.4 (31.4–37.3)
Good	2044 (64.0)	23.1 (21.3–25.0)	10.4 (9.1–11.7)	33.5 (31.5–35.6)
Pearson Chi-square *p*	<0.001	0.734	0.002	0.049
**Quality of life (perception)**				
Very bad	30 (0.9)	16.7 (3.3–30.0)	33.3 (16.5–50.2)	50.0 (32.1–67.9)
Bad	61 (1.9)	23.0 (12.4–33.5)	19.7 (9.7–29.6)	42.6 (30.2–55.0)
Average	522 (16.4)	22.4 (18.8–26.0)	12.1 (9.3–14.9)	34.5 (30.4–38.6)
Good	1054 (33.1)	26.6 (23.9–29.2)	10.0 (8.2–11.8)	36.5 (33.6–39.4)
Very good	1517 (47.6)	22.0 (19.9–24.0)	9.8 (8.3–11.3)	31.8 (29.4–34.1)
Pearson Chi-square *p*	<0.001	0.070	<0.001	0.019

* Response rate: 92.6–98.7%.

**Table 2:** Prevalence of participation in bullying among school-age children in Serbia, 2017.

Variables	Sample * n (%)	Participation in Bullying Prevalence (% and 95% Confidence Interval)		
		At Least Once	Multiple Times	Total
**Individual Characteristics**				
**Gender**				
Boys	1659 (51.4)	9.8 (8.4–11.3)	7.9 (6.6–9.2)	17.7 (15.9–19.6)
Girls	1568 (48.6)	7.3 (6.0–8.6)	3.1 (2.3–4.0)	10.4 (8.9–11.9)
Pearson Chi-square *p*	0.109	0.01	<0.001	<0.001
**Age (years)**				
11–12	872 (27.0)	7.0 (5.3–8.7)	3.8 (2.5–5.1)	10.8 (8.7–12.8)
13–14	939 (29.1)	12.4 (10.2–14.5)	6.2 (4.6–7.7)	18.5 (16.0–21.0)
15–17	1416 (43.9)	7.1 (5.7–8.4)	6.3 (5.0–7.5)	13.3 (11.6–15.1)
Pearson Chi-square *p*	<0.001	<0.001	0.026	<0.001
**School type: grade**				
Primary: grade V	897 (27.8)	7.4 (5.6–9.1)	4.0 (2.7–5.3)	11.4 (9.3–13.4)
Primary: grade VII	928 (28.8)	12.2 (10.1–14.3)	6.0 (4.5–7.6)	18.2 (15.7–20.7)
High school: grade I	1402 (43.4)	7.0 (5.7–8.3)	6.3 (5.0–7.5)	13.3 (11.5–15.0)
Pearson Chi-square *p*	<0.001	<0.001	0.054	<0.001
**Family size**				
2–3 members	392 (12.9)	7.1 (4.6–9.7)	6.1 (3.7–8.5)	13.3 (9.9–16.6)
4–5 members	1790 (58.7)	8.7 (7.3–10.0)	5.2 (4.2–6.3)	13.9 (12.3–15.5)
6–7 members	761 (25.1)	8.7 (6.7–10.7)	4.7 (3.2–6.2)	13.4 (11.0–15.8)
8 and more members	198 (3.2)	9.2 (3.5–14.9)	6.1 (1.4–10.9)	15.3 (8.2–22.4)
Pearson Chi-square *p*	<0.001	0.784	0.762	0.945
**Living with**				
Both parents	2562 (84.5)	8.4 (7.3–9.5)	4.8 (4.0–5.7)	13.2 (11.9–14.5)
One parent	373 (12.3)	7.8 (5.1–10.5)	7.5 (4.8–10.2)	15.3 (11.6–18.9)
One parent and stepfather/mother	59 (1.9)	16.9 (7.4–26.5)	6.8 (0.4–13.2)	23.7 (12.9–34.6)
Relatives/legal guardians	37 (1.2)	8.1 (−0.7–16.9)	8.1 (−0.7–16.9)	16.2 (4.3–28.1)
Pearson Chi-square *p*	<0.001	0.026	0.134	0.091
**Having brothers and sisters**				
None	354 (11.5)	7.6 (4.9–10.4)	4.0 (1.9–6.0)	11.6 (8.2–14.9)
One	1710 (55.5)	8.4 (7.1–9.7)	5.4 (4.3–6.4)	13.7 (12.1–15.4)
Two or more	1014 (32.9)	9.6 (7.8–11.4)	5.4 (4.0–6.8)	15.0 (12.8–17.2)
Pearson Chi-square *p*	<0.001	0.424	0.518	0.268
**Family financial status (perception)**				
Bad	156 (4.9)	10.9 (6.0–15.8)	16.0 (10.3–21.8)	26.9 (20.0–33.9)
Average	993 (31.1)	8.5 (6.7–10.2)	5.4 (4.0–6.8)	13.9 (11.7–16.0)
Good	2048 (64.1)	8.4 (7.2–9.6)	4.7 (3.8–5.7)	13.1 (11.7–14.6)
Pearson Chi-square *p*	<0.001	0.425	<0.001	<0.001
**Quality of life (perception)**				
Very bad	29 (0.9)	6.9 (−2.3–16.1)	34.5 (17.2–51.8)	41.4 (23.5–59.3)
Bad	63 (2.0)	11.1 (3.4–18.9)	14.3 (5.6–22.9)	25.4 (14.6–36.1)
Average	521 (16.3)	11.5 (8.8–14.3)	6.1 (4.1–8.2)	17.7 (14.4–20.9)
Good	1054 (33.1)	10.0 (8.2–11.8)	5.3 (4.0–6.7)	15.3 (13.1–17.4)
Very good	1521 (47.7)	6.5 (5.3–7.7)	4.2 (3.2–5.2)	10.7 (9.2–12.3)
Pearson Chi-square *p*	<0.001	0.002	<0.001	<0.001

* Response rate: 92.8–98.8%.

**Table 3:** Models of participation in fights among school-age children in Serbia, 2017.

Variables	Univariate Logistic Regression		Multivariate Logistic Regression	
	Model 1: At Least Once versus None	Model 2: Multiple Times versus None	Model 1: At Least Once versus None	Model 2: Multiple Times versus None
	OR (95% CI)	OR (95% CI)	OR (95% CI)	OR (95% CI)
**Socio-demographic characteristics**				
**Gender**				
Boys	1	1	1	1
Girls	0.24 *** (0.20–0.29)	0.12 *** (0.09–0.17)	0.23 *** (0.19–0.28)	0.12 *** (0.08–0.16)
**Age (years)**				
11–12	1	1	1	1
13–14	1.35 ** (1.09–1.67)	1.18 (0.85–1.63)	3.15 * (1.27–8.06)	2.39 (0.44–12.9)
15–17	0.92 (0.75–1.13)	1.47 ** (1.11–1.96)	3.99 (0.90–18.4)	3.02 (0.18–50.4)
**School type: grade**				
Primary; grade V	1	1	1	1
Primary; grade VII	1.26 * (1.02–1.56)	1.13 (0.82–1.56)	0.41 (0.16–1.04)	0.44 (0.08–2.37)
High school; grade I	0.89 (0.72–1.08)	1.44 * (1.09–1.92)	0.22 * (0.05–0.98)	0.39 (0.02–6.40)
**Family size**				
2–3 members	1	1		
4–5 members	1.17 (0.89–1.530	0.82 (0.58–1.17)	/	/
6–7 members	1.16 (0.86–1.56)	0.90 (0.60–1.34)	/	/
8 and more members	1.19 (0.69–2.05)	1.47 (0.77–2.82)	/	/
**Living with**				
Both parents	1	1		1
One parent	1.10 (0.85–1.42)	1.04 (0.72–1.51)	/	0.99 (0.64–1.52)
One parent and stepfather/mother	1.40 (0.78–2.52)	1.37 (0.60–3.12)	/	1.52 (0.59–3.88)
Relatives/legal guardians	1.22 (0.53–2.79)	2.89 * (1.25–6.66)	/	2.12 (0.61–7.40)
**Having brothers and sisters**				
None	1	1		
One	0.97 (0.73–1.27)	0.93 (0.64–1.37)	/	/
Two or more	0.99 (0.75–1.33)	0.95 (0.63–1.43)	/	/
**Family financial status (perception)**				
Bad	1	1		1
Average	0.89 (0.59–1.34)	0.45 ** (0.28–0.71)	/	0.56 (0.32–1.00)
Good	0.83 (0.56–1.23)	0.46 *** (0.30–0.71)	/	0.57 (0.32–1.01)
**Quality of life (perception)**				
Very bad	1	1		1
Bad	1.20 (0.34–3.93)	0.51 (0.18–1.45)	/	0.47 (0.12–1.95)
Average	1.03 (0.37–2.88)	0.28 ** (0.12–0.64)	/	0.31 (0.09–1.04)
Good	1.26 (0.45–3.49)	0.24 ** (0.10–0.54)	/	0.24 * (0.07–0.81)
Very good	0.97 (0.35–2.68)	0.22 *** (0.10–0.49)	/	0.21 * (0.06–0.69)
**Perception of school**				
**What do you think about school?**				
I don’t like school at all/I don’t like school too much	1	1	1	1
I love school to some extent	0.76 ** (0.63–0.91)	0.43 *** (0.33–0.55)	0.75 ** (0.62–0.91)	0.51 *** (0.39–0.67)
I love school very much	0.45 *** (0.34–0.58)	0.24 *** (0.16–0.36)	0.43 *** (0.33–0.57)	0.32 *** (0.21–0.49)
**Are school obligations a large burden for you?**				
Not at all/Yes, a little	1	1		1
Yes, quite large	0.97 (0.77–1.21)	1.72 *** (1.29–2.30)	/	1.38 * (1.02–1.86)
Yes, very large	1.21 (0.94–1.57)	3.11 *** (2.31–4.18)	/	2.12 *** (1.54–2.91)
**How do you think your class teacher evaluates your success in relation to others in the class?**				
Below average/average	1	1	1	1
Good	0.96 (0.74–1.26)	0.87 (0.62–1.23)	0.83 (0.66–1.04)	1.03 (0.72–1.47)
Very good	0.76 * (0.60–0.96)	0.57 *** (0.42–0.78)	0.99 (0.76–1.31)	0.81 (0.58–1.11)
**Perception of other students**				
**Students in my class like to be together**				
Disagree	1	1		1
Neither agree nor disagree	0.90 (0.66–1.24)	0.61 * (0.41–0.89)	/	0.81 (0.54–1.22)
Agree	0.88 (0.65–1.18)	0.51 *** (0.36–0.72)	/	0.81 (0.53–1.22)
**Most of the students in my class are friendly and want to help**				
Disagree	1	1		1
Neither agree nor disagree	0.94 (0.69–1.28)	0.54 ** (0.37–0.78)	/	0.58 ** (0.39–0.85)
Agree	0.79 (0.60–1.04)	0.40 *** (0.29–0.54)	/	0.44 *** (0.30–0.64)
**Other students accept me as I am**				
Disagree	1	1		1
Neither agree nor disagree	0.69 (0.45–1.05)	0.60 * (0.36–0.98)	/	0.74 (0.44–1.25)
Agree	0.89 (0.63–1.25)	0.54 ** (0.36–0.80)	/	0.86 (0.55–1.35)
**Perception of professors**				
**I feel that the professors accept me as I am**				
Disagree	1	1		
Neither agree nor disagree	0.72 (0.49–1.05)	0.33 *** (0.22–0.51)	/	0.52 ** (0.34–0.78)
Agree	0.74 (0.53–1.03)	0.27 *** (0.19–0.39)	/	0.41 *** (0.26–0.63)
**I feel that professors take care of me as a person**				
Disagree	1	1	1	
Neither agree nor disagree	1.01 (0.77–1.34)	0.61 ** (0.44–0.84)	1.14 (0.85–1.54)	0.92 (0.63–1.33)
Agree	0.77 * (0.60–0.99)	0.37 *** (0.27–0.50)	0.95 (0.70–1.30)	0.75 (0.50–1.13)
**I have great confidence in my professors**				
Disagree	1	1	1	
Neither agree nor disagree	0.79 (0.62–1.02)	0.49 *** (0.36–0.66)	0.76 (0.61–1.04)	0.61 ** (0.43–0.85)
Agree	0.67 *** (0.53–0.83)	0.32 *** (0.24–0.42)	0.70 * (0.53–0.92)	0.44 *** (0.30–0.64)

* *p* < 0.05; ** *p* < 0.01; *** *p* < 0.001; OR—odds ratio; 95% CI—95% confidence interval.

**Table 4:** Models of participation in bullying among school-age children in Serbia, 2017.

Variables	Univariate Logistic Regression		Multivariate Logistic Regression	
	Model 3: At Least Once versus None	Model 4: Multiple Times versus None	Model 3: At Least Once versus None	Model 4: Multiple Times versus None
	OR (95% CI)	OR (95% CI)	OR (95% CI)	OR (95% CI)
**Socio-demographic characteristics**				
**Gender**				
Boys	1	1	1	1
Girls	0.68 ** (0.53–0.87)	0.36 *** (0.26–0.51)	0.64 ** (0.49–0.83)	0.38 *** (0.27–0.55)
**Age years**				
11–12	1	1	1	1
13–14	1.93 *** (1.40–2.67)	1.79 ** (1.15–2.77)	3.26 * (1.05–10.15)	4.04 * (1.01–16.23)
15–17	1.04 (0.75–1.45)	1.71 * (1.14–2.58)	4.24 (0.64–29.44)	0
**School type: grade**				
Primary; grade V	1	1	1	1
Primary; grade VII	0.97 (0.70–1.34)	1.60 * (1.07–2.38)	0.62 (0.20–1.89)	0.41 (0.10–1.63)
High school; grade I	1.79 *** (1.30–2.47)	1.63 * (1.06–2.51)	0.25 (0.04–1.71)	0
**Family size**				
2–3 members	1	1		
4–5 members	1.22 (0.80–1.86)	0.86 (0.54–1.37)	/	/
6–7 members	1.22 (0.77–1.93)	0.77 (0.45–1.31)	/	/
8 and more members	1.32 (0.60–2.90)	1.02 (0.41–2.58)	/	/
**Living with**				
Both parents	1	1	1	
One parent	0.95 (0.63–1.42)	1.59 (1.04–2.43)	0.96 (0.64–1.44)	/
One parent and stepfather/mother	2.30 * (1.14–4.62)	1.59 (0.56–4.50)	2.35 * (1.16–4.77)	/
Relatives/legal guardians	1.00 (0.30–3.30)	1.73 (0.52–5.75)	1.04 (0.31–3.47)	/
**Having brothers and sisters**				
None	1	1		
One	1.12 (0.73–1.73)	1.39 (0.78–2.48)	/	/
Two or more	1.31 (0.84–2.04)	1.43 (0.78–2.60)	/	/
**Family financial status (perception)**				
Bad	1	1		1
Average	0.66 (0.38–1.15)	0.29 *** (0.17–0.48)	/	0.42 ** (0.23–0.76)
God	0.65 (0.38–1.11)	0.25 *** (0.15–0.40)	/	0.39 ** (0.22–0.71)
**Quality of life (perception)**				
Very bad	1	1		1
Bad	1.27 (0.24–6.70)	0.33 * (0.11–0.94)	/	0.34 (0.10–1.09)
Average	1.19 (0.27–5.27)	0.13 *** (0.05–0.30)	/	0.15 *** (0.06–0.40)
Good	0.99 (0.23–4.39)	0.11 *** (0.05–0.24)	/	0.13 *** (0.05–0.34)
Very good	0.62 (0.14–2.72)	0.08 *** (0.04–0.18)	/	0.11 *** (0.04–0.28)
**Perception of school**				
**What do you think about school?**				
I don’t like school at all/I don’t like school too much	1	1	1	1
I love school to some extent	0.83 (0.63–1.08)	0.54 *** (0.39–0.75)	0.93 (0.70–1.23)	0.76 (0.53–1.08)
I love school very much	0.59 ** (0.40–0.88)	0.41 *** (0.25–0.67)	0.72 (0.48–1.09)	0.62 (0.36–1.05)
**Are school obligations a large burden for you?**				
Not at all/Yes, a little	1	1	1	1
Yes, quite large	1.53 ** (1.13–2.07)	1.47 * (0.98–2.21)	1.41 * (1.03–1.92)	1.24 (0.82–1.88)
Yes, very large	1.58 * (1.11–2.27)	3.53 *** (2.46–5.08)	1.43 * (0.98–2.08)	2.78 *** (1.86–4.08)
**How do you think your class teacher evaluates your success in relation to others in the class?**				
Below average/average	1	1	1	
Good	0.91 (0.62–1.33)	0.49 ** (0.32–0.75)	0.96 (0.66–1.41)	0.55 ** (0.35–0.85)
Very good	0.67 * (0.48–0.94)	0.38 *** (0.27–0.55)	0.74 (0.53–1.05)	0.49 *** (0.33–0.72)
**Perception of other students**				
**Students in my class like to be together**				
Disagree	1	1	1	1
Neither agree nor disagree	0.61 * (0.40–0.94)	0.40 *** (0.25–0.64)	0.79 (0.49–1.19)	0.56 (0.34–0.92)
Agree	0.56 ** (0.38–0.83)	0.36 *** (0.24–0.55)	0.81 (0.52–1.26)	0.69 (0.42–1.13)
**Most of the students in my class are friendly and want to help**				
Disagree	1	1	1	1
Neither agree nor disagree	0.72 (0.48–1.09)	0.48 ** (0.31–0.74)	0.82 (0.53–1.26)	0.53 ** (0.33–0.84)
Agree	0.52 *** (0.36–0.74)	0.26 *** (0.18–0.37)	0.83 * (0.42–0.96)	0.29 *** (0.18–0.46)
**Other students accept me as I am**				
Disagree	1	1	1	1
Neither agree nor disagree	0.75 (0.45–1.28)	0.46 * (0.25–0.85)	0.84 (0.49–1.44)	0.62 (0.32–1.17)
Agree	0.51 ** (0.33–0.78)	0.37 *** (0.23–0.59)	0.66 (0.41–1.06)	0.77 (0.45–1.32)
Perception of professors				
**I feel that the professors accept me as I am**				
Disagree	1	1	1	1
Neither agree nor disagree	0.99 (0.61–1.63)	0.29 *** (0.17–0.48)	1.24 (0.74–2.09)	0.51 ** (0.30–0.84)
Agree	0.57 * (0.36–0.89)	0.21 *** (0.14–0.32)	0.93 (0.55–1.57)	0.42 ** (0.24–0.71)
**I feel that professors take care of me as a person**				
Disagree	1	1	1	1
Neither agree nor disagree	0.57 ** (0.39–0.83)	0.30 *** (0.19–0.46)	0.69 (0.46–1.03)	0.48 ** (0.29–0.80)
Agree	0.46 *** (0.33–0.63)	0.25 *** (0.18–0.36)	0.63 * (0.41–0.96)	0.45 ** (0.28–0.73)
**I have great confidence in my professors**				
Disagree	1	1	1	1
Neither agree nor disagree	0.49 *** (0.34–0.70)	0.39 *** (0.26–0.59)	0.68 ** (0.46–1.02)	0.63 * (0.40–0.99)
Agree	0.48 *** (0.36–0.65)	0.26 *** (0.18–0.38)	0.59 * (0.40–0.89)	0.47 ** (0.29–0.78)

* *p* < 0.05; ** *p* < 0.01; *** *p* < 0.001; OR—odds ratio; 95% CI—95% confidence interval.

## Data Availability

All data are presented in this study. Original data are available on request from IPHS.
